# Preoperative carbohydrate antigen 19-9 as a prognostic biomarker in colorectal cancer with synchronous peritoneal metastasis: multicentre cohort study

**DOI:** 10.1093/bjsopen/zrag060

**Published:** 2026-05-23

**Authors:** Keita Tashiro, Yoshiki Kajiwara, Hideki Ueno, Hirotoshi Kobayashi, Kenjiro Kotake, Kenichi Sugihara, Yoichi Ajioka

**Affiliations:** Department of Surgery, National Defence Medical College Hospital, Saitama, Japan; Department of Surgery, National Defence Medical College Hospital, Saitama, Japan; Department of Surgery, National Defence Medical College Hospital, Saitama, Japan; Department of Surgery, Teikyo University Mizonokuchi Hospital, Kanagawa, Japan; Department of Gastroenterology and Surgery, Sano City Hospital, Tochigi, Japan; Institute of Science Tokyo, Tokyo, Japan; Department of Division of Molecular and Diagnostic Pathology, Niigata University, Niigata, Japan

**Keywords:** colorectal cancer, peritoneal metastasis, CA19-9, carcinoembryonic antigen, prognostic biomarker

## Abstract

**Background:**

Currently, no international guidelines recommend carbohydrate antigen 19-9 (CA19-9) as a biomarker in colorectal cancer in clinical practice, although it has been reported that tumour cells with high affinity for peritoneal cells strongly express CA19-9. The aim of this study was to determine the utility of preoperative serum CA19-9 levels in predicting prognosis in patients with colorectal cancer and synchronous peritoneal metastasis.

**Methods:**

Prognostic analyses were retrospectively performed in patients with colorectal cancer and synchronous peritoneal metastases who received treatment in 16 hospitals between 1991 and 2007 (Cohort 1). The categorization for CA19-9 and carcinoembryonic antigen were established based on the thresholds that minimized Akaike information criterion values for overall survival. A separate validation cohort of patients with synchronous peritoneal metastasis was prospectively registered across 28 institutions between 2012 and 2016 (Cohort 2) to validate these findings.

**Results:**

A total of 689 patients were analysed in Cohort 1 and 144 in the validation Cohort 2. The categorized serum CA19-9 and carcinoembryonic antigen levels were significantly correlated with overall survival (*P* < 0.001 and *P* < 0.001, respectively). The median postoperative overall survival was 19.0 months for patients with a preoperative CA19-9 level < 100 U/ml compared with 4.1 months for those with a preoperative CA19-9 level of ≥ 10 000 U/ml. Using multivariable analysis, serum CA19-9, but not carcinoembryonic antigen, was an independent prognostic factor for overall survival. These results were confirmed in Cohort 2, revealing that the prognostic power of CA19-9 was superior to that of carcinoembryonic antigen.

**Conclusions:**

Preoperative serum CA19-9 outperformed carcinoembryonic antigen as an independent prognostic biomarker in patients with colorectal cancer and peritoneal metastasis.

## Teaser

Overall survival curves based on preoperative serum levels of tumour markers in patients with colorectal cancer and peritoneal metastasis in Cohort 1 were determined. Preoperative serum carbohydrate antigen 19-9 (CA19-9) and carcinoembryonic antigen (CEA) levels were associated with overall survival. However, CA19-9 levels exhibited a stronger, concentration-dependent association with prognosis compared with CEA levels.

## Introduction

Colorectal cancer (CRC) with peritoneal metastasis is associated with a worse prognosis compared with stage IV CRC presenting with metastasis at other sites^1^. These patients are considered in distinct categories according to the Union for International Cancer Control^2^ and American Joint Committee on Cancer^3^ tumour node metastasis staging classifications, as well as the Japanese Classification of Colorectal, Appendiceal, and Anal Carcinoma^4^. However, owing to the inability to detect small metastatic nodules, the diagnosis of peritoneal metastasis of CRC is often challenging despite considerable improvements in imaging technologies^5,6^. Identifying prognostic factors in patients with peritoneal metastasis is important in determining effective therapeutic strategies, including the indication for radical resection.

In Japan, carbohydrate antigen 19-9 (CA19-9) is used as a serum tumour marker for CRC, and the Japanese guideline^7^ for CRC treatment recommends measuring CA19-9. However, according to the 2006 edition of the American Society of Clinical Oncology guidelines^8^, the evidence supporting the use of serum CA19-9 for the screening, diagnosis, staging, surveillance, and treatment monitoring of CRC is insufficient. The clinical utility of serum CA19-9 in CRC^9–13^, particularly its association with peritoneal metastasis^9,12^, has been demonstrated, although previous studies were conducted in single institutions with smaller cohorts. Additionally, one study^14^ reported that tumour cells with an affinity for peritoneal cells strongly expressed CA19-9. Therefore, the aim of this study was to determine the utility of preoperative serum CA19-9 levels in predicting the prognosis of patients with peritoneal metastasis of CRC and using the multi-institutional retrospective and prospective databases of the Japanese Society for Cancer of the Colon and Rectum (JSCCR).

## Methods

The retrospective cohort (Cohort 1) comprised patients who were identified from a database of 3965 patients with stage IV CRC who underwent abdominal surgery between January 1991 and December 2007 across 16 institutions participating in the JSCCR project. The prospective cohort (Cohort 2) comprised a separate group of 150 patients with CRC and synchronous peritoneal metastasis who were prospectively registered across 28 institutes between October 2012 and December 2016 as part of the JSCCR’s multicentre research project titled ‘Grading CRC Peritoneal Metastasis’. To address potential bias and enhance the robustness of the findings, the authors conducted the analyses using these independent databases and validated the results of Cohort 1 in Cohort 2. This study was conducted and reported in accordance with the STROBE guidelines (*Table S1*).

### Patients

The present study included the data of two cohorts of patients with CRC and synchronous peritoneal metastasis. The study size was determined by the number of eligible patients with synchronous peritoneal metastasis from CRC and sufficient clinicopathological information available in the participating databases. Peritoneal metastasis was defined as cases in which the diagnosis was confirmed pathologically or considered to be peritoneal metastasis based on intraoperative findings. Patients with insufficient clinicopathological data were excluded from the analysis. Postoperative follow-up was conducted in accordance with the protocols established by each institution. The patient selection process is shown in *Fig. S1*.

The ethics committees of the JSCCR and all participating institutions approved the study. In Cohort 1, patients were provided with the option to opt out of participation. In Cohort 2, written informed consent was obtained from all patients before enrolment. In both cohorts, informed consent was obtained in accordance with the ethical standards of the revised Declaration of Helsinki. Artificial intelligence tools were not used in the preparation of this manuscript.

### Evaluation of tumour marker levels

The present study compared the utility of preoperative serum CA19-9 and carcinoembryonic antigen (CEA) levels as prognostic factors in patients with CRC and synchronous peritoneal metastasis. *Figure S2* shows the trends in hazard ratios for overall survival (OS) rates based on preoperative serum CA19-9 and CEA levels in Cohort 1. At each given tumour marker level, the hazard ratio for the cohort with the higher value was plotted using the population with the lower value as reference. When the tumour marker levels were plotted on a logarithmic scale, the hazard ratios for both markers showed an almost linear increase with increasing levels. Whilst taking care to establish cut-off values that are easy to use in clinical practice, multiple exponential categorizations were considered for each tumour marker and the classification with the lowest Akaike information criterion (AIC) value was adopted as the tumour marker classification in the present study.

### Statistical analysis

Continuous variables were first assessed for normality using the Shapiro–Wilk test. For comparisons between two independent groups, variables that were non-normally distributed were analysed using the Wilcoxon test. When normality was confirmed, the Levene test was used to evaluate homogeneity of variances; if variances were equal, the Student’s *t*-test was applied, whereas the Welch *t*-test was used when variances were unequal. For comparisons across three or more independent groups, non-normally distributed variables were analysed using the Kruskal–Wallis test, whereas normally distributed variables were assessed using one-way analysis of variance (ANOVA) when variances were equal or the Welch ANOVA when variances were unequal. OS rates after surgery were calculated using the Kaplan–Meier method and compared using the log-rank test. Cox proportional hazards models were used to determine independent prognostic factors for OS. Variables included in the multivariable Cox models were selected from those identified as significant in the univariable analyses. In Cohort 2, the number of variables included in the multivariable model was limited to the number of observed events to avoid model overfitting. Statistical significance was considered at a *P* value of ≤ 0.05 for all analyses. AIC was used to compare the prognostic power of each factor^15^. The model with the most effective outcome prediction and the minimal information loss yielded the lowest AIC value. All statistical analyses were performed using JMP® software version 17 (SAS Institute, Cary, NC, USA).

## Results

Cohort 1 comprised 689 patients who were identified from a database of 3965 patients with stage IV CRC who underwent abdominal surgery. In Cohort 1, 2807 patients without synchronous peritoneal metastasis and 469 patients with insufficient clinicopathological data were excluded. Cohort 2 included 144 of the 150 patients who were registered in the multicentre research project. In Cohort 2, six patients with insufficient data were excluded (*Fig. S1*: CONSORT diagram).

### Patient characteristics

*Table 1* summarizes patient characteristics in both cohorts. The median follow-up period was 13.0 months for Cohort 1 and 21.9 months for Cohort 2. The rate of primary tumour resection was 93.5% (644 of 689) in Cohort 1 and 86.1% (124 of 144) in Cohort 2. The rate was significantly higher in Cohort 1 (χ^2^ test: *P* = 0.003). In Cohort 1, among patients who underwent resection without central vascular ligation (CVL), 92 patients (63.4%) had distant metastases. Of the remaining 53 patients, 32 cases (60%) had 11 or more peritoneal metastatic lesions. In contrast, among patients who underwent resection with CVL, 265 patients (53.1%) had distant metastases, and, of the remaining 234 patients, 86 (36.8%) had 11 or more peritoneal metastatic lesions. Both comparisons showed statistically significant differences between the groups with and without CVL (χ^2^ test: *P* = 0.027 and *P* = 0.002, respectively). In Cohort 2, distant metastases were observed in 18 of 22 patients (82%) who underwent resection without CVL, and all 4 of the remaining patients had 11 or more peritoneal metastatic lesions. Conversely, in the group that underwent resection with CVL, 50 of 102 patients (49.0%) had distant metastases, and, of the remaining 52 patients, 19 (36.5%) had 11 or more peritoneal metastatic lesions. These differences were statistically significant (χ^2^ test: *P* = 0.005 and *P* = 0.013, respectively).

**Table 1:** Background patient characteristics

	Cohort 1	Cohort 2
	*n* = 689	*n* = 144
Age (years), mean (range)	62 (14–86)	66 (30–89)
**Sex**		
Male	354	82
Female	335	62
**Location**		
Colon	510	118
Rectum	179	26
**Type of primary tumour surgery**		
No resection	45	20
Resection without CVL	145	22
Resection with CVL	499	102
**Histologic type**		
tub	514	113
Others	175	31
**T category**		
T1–3	223	11
T4	415	113
Unknown	51	20
**N category**		
N0/N1	303	71
N2	341	53
Unknown	45	20
**Lymphatic invasion**		
Negative	39	17
Positive	606	107
Unknown	44	20
**Venous invasion**		
Negative	67	9
Positive	578	114
Unknown	44	21
**Distant metastasis**		
Negative	484	61
Positive	202	83
Unknown	3	0
**Number of peritoneal metastatic lesions**		
1	152	19
2–10	237	53
≥ 11	300	72
Preoperative CA19-9 level (U/ml), median (i.q.r.)	54.0 (13.0–277.7)	49.0 (10.0–337.3)
Preoperative CEA level (ng/ml), median (i.q.r.)	19.8 (5.4–85.3)	22.4 (5.2–145.9)
3-year OS rate (%)	16.4%	31.6%

Values are *n* (%) unless otherwise stated. CVL, central vascular ligation; tub, tubular adenocarcinoma; CA19-9, carbohydrate antigen 19-9; i.q.r., interquartile range; CEA, carcinoembryonic antigen; OS, overall survival.

### Establishing categories for tumour marker values

The AIC values for OS were evaluated across multiple exponential categorizations of each tumour marker (*Fig. S3*). In all cases, the AIC values were lower for CA19-9 than for CEA. The AIC for serum CA19-9 was minimized (AIC 6649) when the marker levels were categorized as < 100, ≥ 100, and < 1000; ≥ 1000 and < 10,000; and ≥ 10 000 U/ml. The AIC for serum CEA was minimized (AIC 6687) when the marker levels were categorized as < 4, ≥ 4, and < 40; ≥ 40 and < 400; and ≥ 400 ng/ml. These categorizations were used for the categorizations of patients according to serum tumour marker levels.

### Univariable and multivariable analyses of OS in Cohort 1

**Fig. 1:**
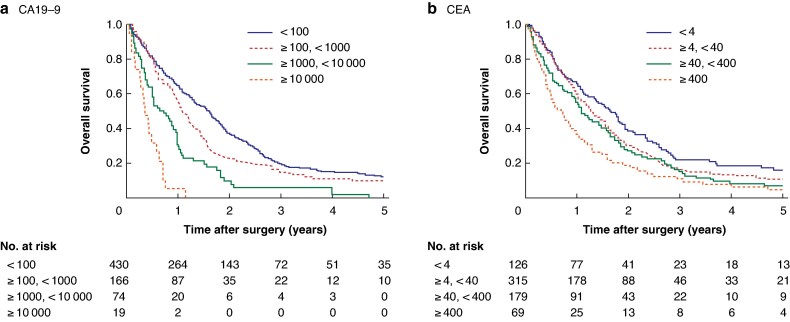
Kaplan–Meier plots showing overall survival curves based on preoperative serum levels of tumour markers in patients with colorectal cancer and peritoneal metastasis in Cohort 1 **a** Preoperative serum CA19-9 levels, **b** preoperative serum CEA levels. Preoperative serum CA19-9 and CEA levels were associated with overall survival. However, CA19-9 levels exhibited a stronger, concentration-dependent association with prognosis compared with CEA levels. The median survival times were 19.0, 13.0, 7.8, and 4.1 months in patients with preoperative CA19-9 levels of < 100, ≥ 100–< 1000, ≥ 1000–< 10,000, and ≥ 10 000 U/ml, respectively (*P* < 0.001), and 19.9, 15.3, 13.0, and 8.5 months in patients with preoperative CEA levels of < 4, ≥ 4–< 40, ≥ 40–< 400, and ≥ 400 ng/ml, respectively (*P* < 0.001). CA19-9, carbohydrate antigen; CEA, carcinoembryonic antigen.

*Figure 1* shows the OS curves based on preoperative serum CA19-9 and CEA levels in Cohort 1. Preoperative serum CA19-9 and CEA levels were significantly correlated with OS (*P* < 0.0001 for both). *Table 2* shows the results of univariable and multivariable analyses of OS in Cohort 1. Briefly, age, type of primary tumour surgery, N category, distant metastasis, number of peritoneal metastatic lesions, and preoperative serum CA19-9 and CEA levels were significantly correlated with OS. Preoperative serum CA19-9 levels, type of primary tumour surgery, advanced age, high degree of lymph node metastasis, presence of distant metastases, and large number of peritoneal metastatic lesions were independent risk factors for OS in patients with CRC and synchronous peritoneal metastasis. Several of these independent factors showed significant associations with preoperative CA19-9 levels. These included the type of primary surgery (mean CA19-9 level: no resection 3465.5 U/ml; resection without CVL 2629.5 U/ml; resection with CVL 1999.9 U/ml; *P* < 0.001), N category (N0/N1 1700.3 U/ml *versus* N2 2555.6 U/ml; *P* = 0.049), distant metastasis (negative 281.1 U/ml *versus* positive 3802.1 U/ml; *P* < 0.001), and the number of peritoneal metastatic lesions (1 lesion 1893.6 U/ml; 2–10 lesions 2272.2 U/ml; ≥ 11 lesions 2362.8 U/ml; *P* = 0.001).

**Table 2:** Univariable and multivariable analyses for overall survival (Cohort 1)

	Cohort 1 patients (*n* = 689)	2-year OS	Univariable analysis		Multivariable analysis	
			HR	P*	HR	P*
**Age (years)**						
< 80	639	30.9%	1.00		1.00	
≥ 80	50	12.8%	1.67 (1.23–2.27)	< 0.001	1.66 (1.18–2.32)	0.003
**Sex**						
Male	354	26.4%	1.00		–	–
Female	335	32.6%	0.87 (0.74–1.03)	0.1	–	–
**Location**						
Colon	510	28.1%	1		–	–
Rectum	179	33.2%	0.87 (0.72–1.05)	0.135	–	–
**Type of primary tumour surgery**						
No resection	45	5.7%	1.00		1.00	
Resection without CVL	145	15.7%	0.54 (0.37–0.79)	0.001	0.27 (0.11–0.61)	0.002
Resection with CVL	499	34.9%	0.32 (0.23–0.45)	< 0.001	0.18 (0.08–0.41)	< 0.001
**Histologic type**						
tub	514	31.0%	1.00		–	–
Others	175	24.9%	1.16 (0.96–1.40)	0.114	–	–
**T category**						
T1–T3	223	32.5%	1.00		–	–
T4	415	30.1%	1.04 (0.87–1.23)	0.679	–	–
**N category**						
N0/N1	303	36.6%	1.00		1.00	
N2	341	25.7%	1.38 (1.17–1.63)	< 0.001	1.35 (1.13–1.60)	< 0.001
**Distant metastasis**						
Negative	308	37.3%	1.00		1.00	
Positive	381	22.9%	1.58 (1.34–1.86)	< 0.001	1.45 (1.21–1.74)	< 0.001
**No. peritoneal metastatic lesions**						
1	152	45.2%	1.00		1.00	
2–10	237	32.0%	1.42 (1.13–1.79)	0.002	1.45 (1.15–1.82)	0.002
≥ 11	300	18.7%	2.04 (1.64–2.54)	< 0.001	1.78 (1.42–2.24)	< 0.001
**CA19-9 (U/ml)**						
< 100	430	36.7%	1.00		1.00	
≥ 100, < 1000	166	22.4%	1.27 (1.05–1.55)	0.015	1.01 (0.81–1.26)	0.918
≥ 1000, < 10 000	74	9.8%	2.28 (1.74–2.99)	< 0.001	1.69 (1.24–2.30)	< 0.001
≥ 10 000	19	0%	5.75 (3.57–9.25)	< 0.001	4.03 (2.32–6.99)	< 0.001
**CEA (ng/ml)**						
< 4	126	38.3%	1.00		1.00	
≥ 4, < 40	315	29.5%	1.32 (1.05–1.67)	0.019	1.21 (0.95–1.55)	0.128
≥ 40, < 400	179	27.4%	1.48 (1.15–1.91)	0.003	1.20 (0.91–1.59)	0.206
≥ 400	69	16.9%	2.11 (1.54–2.89)	**<** 0.001	1.35 (0.94–1.96)	0.108

Values in parentheses are 95% confidence intervals. OS, overall survival; HR, hazard ratio; –, not applicable; CVL, central vascular ligation; tub, tubular adenocarcinoma; CA19-9, carbohydrate antigen 19-9; CEA, carcinoembryonic antigen. *P value were calculated using univariable and multivariable Cox proportional hazards models.

### Validation of prognostic factors for CRC with peritoneal metastasis in Cohort 2

**Fig. 2:**
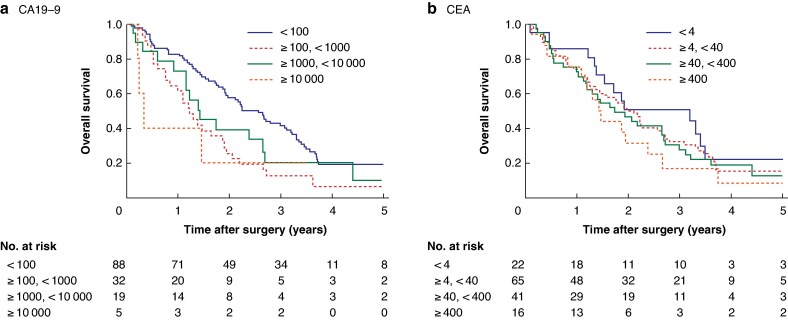
Kaplan–Meier plots showing overall survival curves based on preoperative serum levels of tumour markers in patients with colorectal cancer and peritoneal metastasis in Cohort 2 **a** Preoperative serum CA19-9 levels, **b** preoperative serum CEA levels. The prognosis was better in patients with lower preoperative serum CA19-9 levels than in those with higher preoperative serum CA19-9 levels, whereas such an association was not observed for preoperative serum CEA levels. Similar to that observed in Cohort 1, serum CA19-9 levels tended to stratify prognosis in a concentration-dependent manner. The median survival times were 30.1, 14.6, 16.8, and 3.8 months in patients with preoperative CA19-9 levels of < 100, ≥ 100–< 1000, ≥ 1000–< 10,000, and ≥10 000 U/m, respectively (*P* = 0.010), and 38.4, 23.7, 20.9, and 17.3 months in patients with preoperative CEA levels of < 4, ≥ 4–< 40, ≥ 40–< 400, and ≥ 400 ng/ml, respectively (*P* = 0.512). CA19-9, carbohydrate antigen; CEA, carcinoembryonic antigen.

*Figure 2* shows the OS curves based on preoperative serum CA19-9 and CEA levels in Cohort 2. As for Cohort 1, the preoperative serum CA19-9 level was significantly correlated with OS (*P* = 0.010). However, a similar correlation was not observed for CEA (*P* = 0.512). In Cohort 2, the AIC value was lower for serum CA19-9 than for serum CEA (966 *versus* 974).

The independent prognostic factors identified in Cohort 1 were validated in the multivariable analysis of OS in Cohort 2 (*Table 3*), which confirmed that type of primary tumour surgery, N category, and preoperative serum CA19-9 level were independent prognostic factors. However, the preoperative serum CEA level was not an independent prognostic factor in patients with CRC and synchronous peritoneal metastasis (*Table S2*).

**Table 3:** Multivariable analysis of overall survival in Cohort 2

	Cohort 2 patients (*n* = 144)	2-year OS	Multivariable analysis	
			HR	P*
**Age (years)**				
< 80	127	49.8%	1.00	
≥ 80	17	29.4%	1.64 (0.93–2.91)	0.090
**Type of primary tumour surgery**				
No resection	20	15.0%	1.00	
Resection without CVL	22	15.0%	0.53 (0.27–1.05)	0.068
Resection with CVL	102	55.9%	0.41 (0.23–0.73)	0.002
**N category**				
N0/N1	81	55.4%	1.00	
N2	63	35.5%	1.86 (1.25–2.77)	0.002
**Distant metastasis**				
Negative	61	60.6%	1.00	
Positive	83	36.6%	1.03 (0.65–1.65)	0.890
**No. peritoneal metastatic lesions**				
1	19	66.7%	1.00	
2–10	53	55.0%	1.14 (0.58–2.24)	0.695
≥ 11	72	35.4%	1.85 (0.97–3.56)	0.063
**CA19-9 (U/ml)**				
< 100	88	57.5%	1.00	
≥ 100, < 1000	32	25.8%	1.84 (1.11–3.06)	0.019
≥ 1000, < 10 000	19	39.3%	1.39 (0.75–2.58)	0.300
≥ 10 000	5	20.2%	1.51 (0.53–4.36)	0.442

Values in parentheses are 95% confidence intervals. OS, overall survival; HR, hazard ratio; CA19-9, carbohydrate antigen 19-9; CVL, central vascular ligation. *P value were calculated using multivariable Cox proportional hazards models.

## Discussion

This study demonstrates the utility of preoperative serum CA19-9 levels in predicting the prognosis of patients with CRC and synchronous peritoneal metastasis using two distinct data sets from retrospective and prospective cohorts. Previous studies^9,12^ evaluating preoperative serum CA19-9 levels in patients with CRC and synchronous peritoneal metastasis included small cohorts and were insufficient to demonstrate clearly its prognostic utility. In the present study, the aim was to overcome this by using a larger cohort and validation with prospectively collected data.

CA19-9 was initially isolated from spleen cells of a mouse immunized with a human colon carcinoma cell line^16,17^, and Herlyn *et al*.^18^ were the first to report the potential utility of serum CA19-9 in colorectal, gastric, and pancreatic carcinoma. Although CA19-9 is recognized as a clinically useful marker in pancreatic adenocarcinoma^19^, its clinical value in CRC is considered limited, as indicated by several international guidelines^8,20,21^. This may be attributed to the lower tumour specificity of CA19-9 compared with CEA^17,22^, a view supported by reports^23^ showing false positivity rates of 15–30% when serum CA19-9 is used as a diagnostic marker in patients with non-neoplastic disease of the pancreas, liver, and biliary tract. Elevated serum CA19-9 levels have also been reported in patients with diabetes or non-malignant gynaecologic and respiratory diseases^24^. Furthermore, given that CA19-9 is a carbohydrate antigen comprising a sialylated form of Lewis A, a blood antigen, serum CA19-9 levels lead to false-negative results in Lewis-negative individuals, even in those with carcinoma^12^.

Although factors unrelated to cancer can impact serum CA19-9 levels, which could introduce uncertainty regarding its clinical utility for CRC, the analysis of retrospective and prospective cohorts in the present study allowed identification of preoperative serum CA19-9 level, N category, and number of peritoneal metastatic lesions as independent prognostic factors in patients with CRC and peritoneal metastasis. Serum CA19-9 levels may be better in stratifying prognosis compared with the other clinicopathologic factors, as shown in *Table 2*. However, serum CEA levels were not an independent prognostic factor in the present study. Overall, these results indicate that preoperative serum CA19-9 levels may be superior to serum CEA levels as a prognostic marker for CRC with peritoneal metastasis.

Several clinical studies have reported the association of CA19-9 in serum and peritoneal fluid and peritoneal metastasis in CRC. In one study^25^, elevated postoperative serum CA19-9 levels were associated with peritoneal recurrence. Other studies^26,27^ have suggested that CA19-9 levels in peritoneal fluid are associated with peritoneal metastasis. Whilst the mechanism underlying the association of CA19-9 specifically with peritoneal metastasis remains unclear, two factors should be considered. First, CA19-9 is strongly expressed in tumour cells that adhere to the peritoneum. By immunocytologic evaluation, Schott *et al*.^14^ demonstrated that peritoneal tumour cells isolated from patients with colorectal or gastric cancer exhibited strong binding affinity for anti-CA19-9 antibodies. Specifically, the tumour cells collected from the peritoneal lavage fluid bound to anti-CA19-9 antibodies at rates of approximately 20 and 40% in samples from patients with CRC and gastric cancer, respectively; these rates were higher than that observed for other antibodies, including those targeting CEA, membrane antigens, and cytokeratin. Additionally, CA19-9 expression in tumour cells has been reported to correlate with tumour cell adhesion^28^. Second, CA19-9 may be associated with hypoxic tumour microenvironments. Peritoneal metastasis is considered to arise from individual tumour cells or small clusters that detach from the primary tumour before undergoing epithelial–mesenchymal transition and subsequently proliferating within the peritoneal cavity^29^. In contrast to haematogenous metastasis, peritoneal metastasis arises in a hypoxic environment that induces the expression of hypoxia-inducible factor 1 alpha and angiogenesis-related genes, including the vascular endothelial growth factor family members^30,31^. The reported association of hypoxia-inducible factor 1 alpha expression in tumour cells with serum CA19-9 levels in patients with intrahepatic cholangiocarcinoma^32^ suggests that a high serum CA19-9 level may reflect tumour hypoxia. Based on these findings, the authors propose that CA19-9 may serve as a clinically meaningful biomarker in CRC with a strong tendency towards peritoneal metastasis and should be considered as a diagnostic and prognostic marker in such cases.

The present study had several limitations. First, Cohort 1 included an extended time period of 1991–2007, during which several advances in CRC chemotherapy were introduced. The prognosis of CRC began significantly improving in the 2000s, following the introduction of highly effective oxaliplatin and irinotecan^33,34^. In fact, the overall 3- and 5-year OS rates were 16.4% and 9.7% in Cohort 1 and 31.6% and 15.0% in Cohort 2, respectively. These findings highlight the differences in chemotherapy practices between the two cohorts. Second, the present study findings could not fully reflect the impact of chemotherapy on outcomes, as the Cohort 1 database lacked sufficient information regarding chemotherapy. Therefore, the prognostic factors identified in Cohort 1 were validated using the data from Cohort 2 to align better with the current clinical practices. In contrast, only 31 cases in Cohort 2 achieved R0 resection, and this number is not sufficient to evaluate adequately the association between the CA19-9 categories used in this study and recurrence after R0 resection. Thirdly, although R0 resection of CRC including synchronous peritoneal metastatic lesions is strongly associated with prognosis after surgery^35–37^, this study did not include the feasibility of R0 resection as an analytical factor. The database used for Cohort 1 did not contain information on whether R0 resection of peritoneal metastatic lesions was performed during primary tumour surgery. To address the lack of information, data were included on whether the primary tumour was resected and central lymph node dissection was performed. Overall, resection with CVL was selectively performed in patients for whom curative treatment was considered feasible, considering the extent of distant and peritoneal metastases. Patients without primary tumour resection were presumed to not have undergone resection of peritoneal metastasis also. Conversely, patients who underwent CVL were considered more likely to have undergone resection of peritoneal metastatic lesions as well. Receiver operating characteristic (ROC) curves are commonly used to determine optimal cut-off values for continuous variables such as tumour markers. However, when cut-off values for CEA and CA19-9 were derived using ROC analysis, they varied substantially depending on the postoperative period analysed. In the present study, to enhance clinical applicability, multiple cut-off values were established that are straightforward to use in routine practice and the most appropriate model based on the AIC was selected. Accordingly, although the cut-off values used in this study do not necessarily achieve the highest prognostic discriminatory ability, they could provide clinically practical and user-friendly values for clinicians. Despite these limitations, the present study provides clinically valuable insights by demonstrating that preoperative CA19-9 levels are an independent prognostic factor in patients with CRC and synchronous peritoneal metastasis and that markedly elevated CA19-9 levels are associated with a poor prognosis. In such patients, it may be preferable to avoid overly invasive procedures, such as complete resection of synchronous peritoneal metastases combined with extensive resection of other organs or the addition of perioperative intraperitoneal chemotherapy, as these approaches may be associated with a high risk of postoperative complications and a reduction in quality of life^38,39^. Therefore, serum CA19-9 levels may be valuable in guiding the selection of the surgical approach for patients with CRC and peritoneal metastasis.

Analyses of two independent cohorts suggest that preoperative serum CA19-9 levels may have clinical value in patients with CRC presenting with synchronous peritoneal metastasis. However, as both cohorts were derived from Japanese multicentre data sets, the generalizability of these findings to non-Japanese populations should be interpreted with caution. Further studies using independent data sets are required to determine whether serum CA19-9 levels are a reliable prognostic stratification marker for peritoneal metastasis in CRC patients. These should include clinical factors not examined in the present study, such as chemotherapy and R0 resection, as well as patients with metachronous or recurrent peritoneal metastasis.

## Supplementary Material

zrag060_Supplementary_Data

## Data Availability

The data that support the findings of this study are available from the JSCCR upon reasonable request and with permission from the relevant committees.
