# Supplementary material for: Preoperative carbohydrate antigen 19-9 as a prognostic biomarker in colorectal cancer with synchronous peritoneal metastasis: multicentre cohort study
Source: BJS Open. 2026 May 23;10(3):zrag060. doi: 10.1093/bjsopen/zrag060 (PMC13198021; doi:10.1093/bjsopen/zrag060)
Supplement: zrag060_Supplementary_Data [file zrag060_supplementary_data.docx]

**Preoperative CA19-9 as a prognostic biomarker in colorectal cancer with synchronous peritoneal metastasis: multicentre cohort study**

Keita Tashiro, MD^1^, Yoshiki Kajiwara, MD, PhD^1^, Hideki Ueno, MD, PhD^1^, Hirotoshi Kobayashi, MD, PhD^2^, Kenjiro Kotake, MD, PhD^3^, Kenichi Sugihara, MD, PhD^4^ and Yoichi Ajioka, MD, PhD^5^

^1^ Department of Surgery, National Defence Medical College Hospital, Saitama, Japan

^2^ Department of Surgery, Teikyo University Mizonokuchi Hospital, Kanagawa, Japan

^3^ Department of Gastroenterology and Surgery, Sano City Hospital, Tochigi, Japan

^4^ Institute of Science Tokyo, Tokyo, Japan

^5^ Department of Division of Molecular and Diagnostic Pathology, Niigata University, Niigata, Japan

**Corresponding author.**

Yoshiki Kajiwara

Department of Surgery, National Defence Medical College Hospital

3‑2 Namiki, Tokorozawa, Saitama 359‑0042, Japan

Tel: +81-4-2995-1637; Fax: +81-4-2996-5205; E-mail: ykaji@ndmc.ac.jp

**Supplementary Materials - Index**

| **Supplementary Methods** |  |
| --- | --- |
| Not applicable |  |
| **Supplementary Results** |  |
| Not applicable |  |
| **Supplementary Appendixes** |  |
| Supplementary Table 1 – STROBE checklist for cohort studies | *page 2*–*3* |
| **Supplementary Figures and Tables** |  |
| Supplementary Table 2 – Multivariate analysis of overall survival in Cohort 2, including CEA | *page 4* |
| Supplementary Fig. 1 – CONSORT diagram | *page 5* |
| Supplementary Fig. 2 – trends in hazard ratios for overall survival based on preoperative  serum CA19-9 and CEA levels in Cohort 1. | *page 6* |
| Supplementary Fig. 3 – Akaike information criterion values for overall survival were evaluated  across multiple exponential categorizations of each tumour marker | *page 7* |
| Supplemental legends | *page 8* |
| **References** |  |
| Not applicable |  |

**Supplementary Appendixes**

Supplementary Table 1. STROBE checklist for cohort studies

| **Item** | No | Recommendation | Page No |
| --- | --- | --- | --- |
| **Title and abstract** |  |  |  |
|  | 1 | (*a*) Indicate the study’s design with a commonly used term in the title or the abstract | 1–2 |
|  |  | (*b*) Provide in the abstract an informative and balanced summary of what was done and what was found | 2–3 |
| Introduction | | |  |
| Background/rationale | 2 | Explain the scientific background and rationale for the investigation being reported | 4–5 |
| Objectives | 3 | State specific objectives, including any prespecified hypotheses | 4–5 |
| Methods | | |  |
| Study design | 4 | Present key elements of study design early in the paper | 6 |
| Setting | 5 | Describe the setting, locations, and relevant dates, including periods of recruitment, exposure, follow-up, and data collection | 6 |
| Participants | 6 | (*a*) Give the eligibility criteria, and the sources and methods of selection of participants. Describe methods of follow-up | 6 |
|  |  | (*b*) For matched studies, give matching criteria and number of exposed and unexposed | Not applicable |
| Variables | 7 | Clearly define all outcomes, exposures, predictors, potential confounders, and effect modifiers. Give diagnostic criteria, if applicable | 6–8 |
| Data sources/ measurement | 8* | For each variable of interest, give sources of data and details of methods of assessment (measurement). Describe comparability of assessment methods if there is more than one group | 6–8 |
| Bias | 9 | Describe any efforts to address potential sources of bias | 6 |
| Study size | 10 | Explain how the study size was arrived at | 6 |
| Quantitative variables | 11 | Explain how quantitative variables were handled in the analyses. If applicable, describe which groupings were chosen and why | 7,8 |
| Statistical methods | 12 | (*a*) Describe all statistical methods, including those used to control for confounding | 7,8 |
|  |  | (*b*) Describe any methods used to examine subgroups and interactions | Not applicable |
|  |  | (*c*) Explain how missing data were addressed | 6 |
|  |  | (*d*) If applicable, explain how loss to follow-up was addressed | Not applicable |
|  |  | (*e*) Describe any sensitivity analyses | Not applicable |
| Results | | |  |
| Participants | 13* | (a) Report numbers of individuals at each stage of study—eg numbers potentially eligible, examined for eligibility, confirmed eligible, included in the study, completing follow-up, and analysed | 9 |
|  |  | (b) Give reasons for non-participation at each stage | 9 |
|  |  | (c) Consider use of a flow diagram | 9 |
| Descriptive data | 14* | (a) Give characteristics of study participants (eg demographic, clinical, social) and information on exposures and potential confounders | 9,10 |
|  |  | (b) Indicate number of participants with missing data for each variable of interest | 9 |
|  |  | (c) Summarise follow-up time (eg, average and total amount) | 9 |
| Outcome data | 15* | Report numbers of outcome events or summary measures over time | 9–12 |
| Main results | 16 | (*a*) Give unadjusted estimates and, if applicable, confounder-adjusted estimates and their precision (eg, 95% confidence interval). Make clear which confounders were adjusted for and why they were included | 10–12 |
|  |  | (*b*) Report category boundaries when continuous variables were categorized | 10 |
|  |  | (*c*) If relevant, consider translating estimates of relative risk into absolute risk for a meaningful time period | Not applicable |
| Other analyses | 17 | Report other analyses done—eg analyses of subgroups and interactions, and sensitivity analyses | Not applicable |
| Discussion | | |  |
| Key results | 18 | Summarise key results with reference to study objectives | 13 |
| Limitations | 19 | Discuss limitations of the study, taking into account sources of potential bias or imprecision. Discuss both direction and magnitude of any potential bias | 15–17 |
| Interpretation | 20 | Give a cautious overall interpretation of results considering objectives, limitations, multiplicity of analyses, results from similar studies, and other relevant evidence | 13–17 |
| Generalisability | 21 | Discuss the generalisability (external validity) of the study results | 17 |
| Other information | | |  |
| Funding | 22 | Give the source of funding and the role of the funders for the present study and, if applicable, for the original study on which the present article is based | 29 |

*Give information separately for exposed and unexposed groups.

STROBE, the Reporting of Observational Studies in Epidemiology

**Supplementary Figures and Tables**

Supplementary Table 2. Multivariate analysis of overall survival in Cohort 2, including CEA

| Clinicopathological factors | Categories | n | Multivariate analysis | | |
| --- | --- | --- | --- | --- | --- |
|  |  |  | HR (95% CI) | | *P* value |
| Age (y) | <80 | 127 | 1 | |  |
|  | ≥80 | 17 | 1.65 (0.92–2.97) | | 0.092 |
| Type of primary tumor surgery | No resection | 20 | 1 | |  |
|  | Resection without CVL | 22 | 0.56 (0.28–1.13) | | 0.104 |
|  | Resection with CVL | 102 | 0.41 (0.23–0.73) | | **0.003** |
| N category | N0/1 | 81 | 1 | |  |
|  | N2 | 63 | 1.90 (1.27–2.84) | | **0.002** |
| Distant metastasis | Negative | 61 | 1 | |  |
|  | Positive | 83 | 1.02 (0.63–1.68) | | 0.926 |
| Number of peritoneal metastatic lesions | 1 | 19 | 1 | |  |
|  | 2–10 | 53 | 1.14 (0.58–2.23) | | 0.713 |
|  | ≥11 | 72 | 1.84 (0.96–3.54) | | 0.068 |
| CA19-9 | <100 | 88 | | 1 |  |
| (U/mL) | ≥100, <1000 | 32 | | 1.93 (1.15–3.24) | **0.013** |
|  | ≥1000, <10,000 | 19 | | 1.41 (0.73–2.72) | 0.302 |
|  | ≥10,000 | 5 | | 1.75 (0.58–5.27) | 0.351 |
| CEA | <4 | 22 | | 1 |  |
| (ng/mL) | ≥4, <40 | 65 | | 0.96 (0.51–1.80) | 0.892 |
|  | ≥40, <400 | 41 | | 0.82 (0.41–1.67) | 0.593 |
|  | ≥400 | 16 | | 1.27 (0.55–2.92) | 0.571 |
| OS, overall survival; HR, hazard ratio; CI, confidence interval;  CA19-9, carbohydrate antigen 19-9; CEA, carcinoembryonic antigen;  CVL, central vascular ligation | | | | | |

**
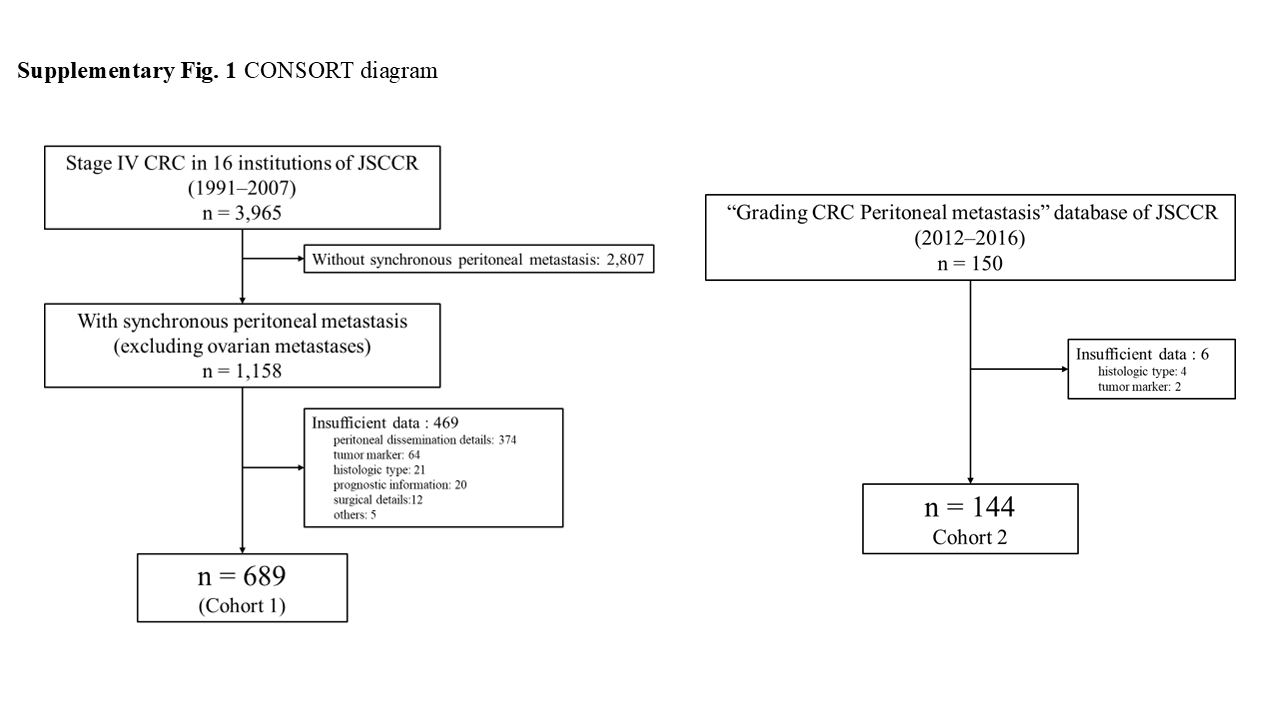
**


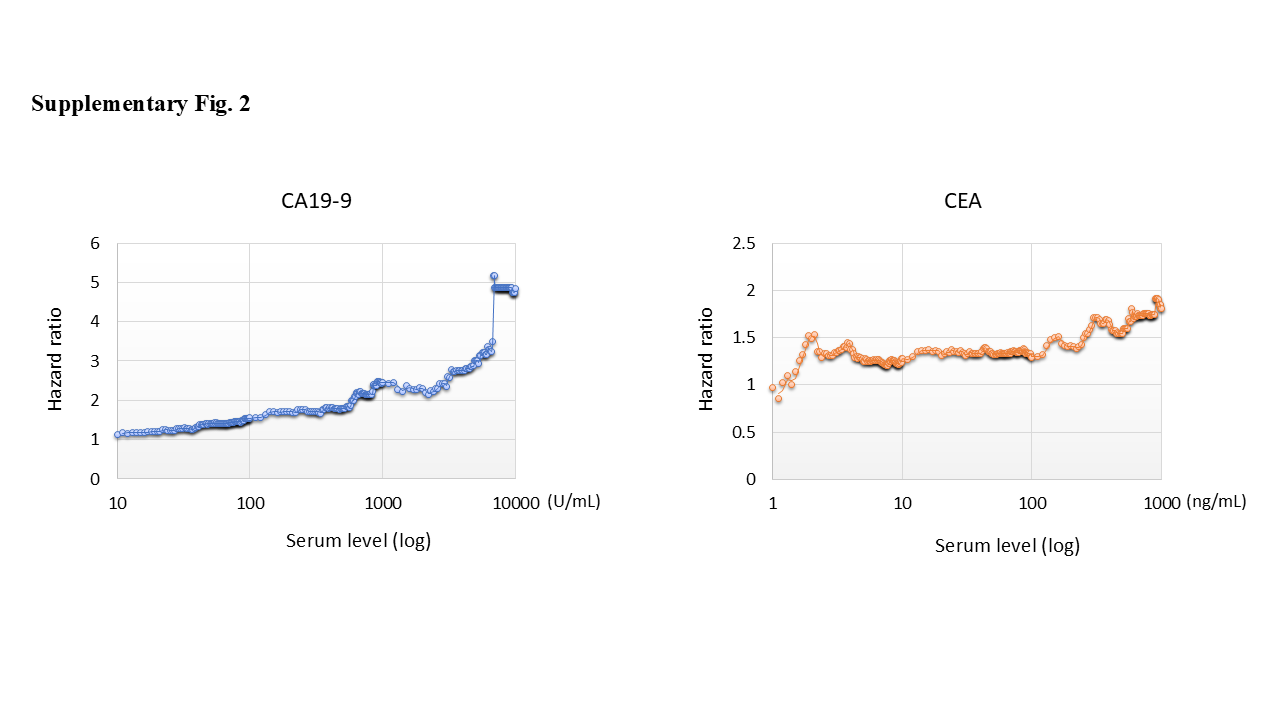


**
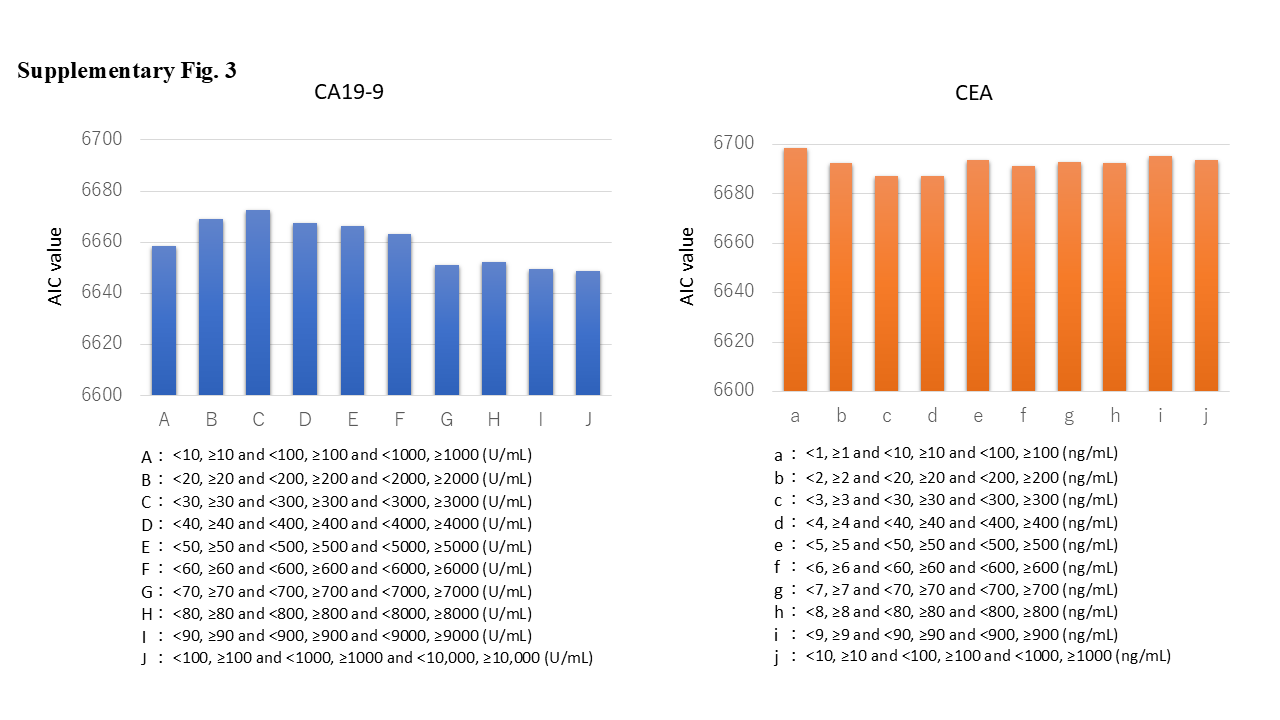
**

**Supplemental legends**

**Supplementary Table 2.** Multivariate analysis of overall survival in Cohort 2, including CEA. The independent prognostic factors identified in Cohort 1 were validated in the multivariate analysis of overall survival in Cohort 2 including preoperative serum CEA levels. CEA level was not an independent prognostic factor in patients with and synchronous peritoneal metastasis.

**Supplementary Fig 1.** Consort diagram for each cohort. Cohort 1 (retrospective) comprised 689 patients, and Cohort 2 (prospective) comprised 144 patients.

**Supplementary Fig 2.** The trends in hazard ratios for overall survival based on preoperative serum CA19-9 and CEA levels in Cohort 1. At each given tumor marker level, the hazard ratio for the cohort with the higher value was plotted using the population with the lower value as reference. When the tumor marker levels were plotted on a logarithmic scale, the hazard ratios for both markers showed an almost linear increase with increasing levels.

**Supplementary Fig 3.** The Akaike information criterion (AIC) values for overall survival were evaluated across multiple exponential categorizations of each tumor marker. In all cases, the AIC values were lower for CA19-9 than for CEA. The AIC for serum CA19-9 was minimized (AIC, 6649) when the marker levels were categorized as <100, ≥100 and <1000, ≥1000 and <10,000, and ≥10,000 U/mL. The AIC for serum CEA was minimized (AIC, 6687) when the marker levels were categorized as <4, ≥4 and <40, ≥40 and <400, and ≥400 ng/mL.
